# Interaction of Phospholipid, Cholesterol, Beta-Carotene, and Vitamin C Molecules in Liposome-Based Drug Delivery Systems: An *In Silico* Study

**DOI:** 10.1155/2023/4301310

**Published:** 2023-01-04

**Authors:** D. Hudiyanti, V. N. R. Putri, Y. Hikmahwati, S. M. Christa, P. Siahaan, D. S. B. Anugrah

**Affiliations:** ^1^Department of Chemistry, Faculty of Science and Mathematics, Diponegoro University, Professsor Soedarto Street, Semarang 50275, Central Java, Indonesia; ^2^Chemistry Program, Faculty of Science and Mathematics, Diponegoro University, Professor Soedarto Street, Semarang 50275, Central Java, Indonesia; ^3^Department of Biotechnology, Faculty of Biotechnology, Atma Jaya Catholic University of Indonesia, BSD Campus, Tangerang 15345, Indonesia

## Abstract

This paper investigates the interaction within a liposome-based drug delivery system *in silico*. Results confirmed that phospholipids, cholesterol, beta-carotene, and vitamin C in the liposome structures interact noncovalently. The formation of noncovalent interactions indicates that the liposomal structures from phospholipid molecules will not result in chemical changes to the drug or any molecules encapsulated within. Noncovalent interactions formed include (i) moderate-strength hydrogen bonds with interaction energies ranging from −73.6434 kJ·mol^−1^ to −45.6734 kJ·mol^−1^ and bond lengths ranging from 1.731 Å to 1.827 Å and (ii) van der Waals interactions (induced dipole-induced dipole and induced dipole-dipole interactions) with interaction energies ranging from −4.4735 kJ·mol^−1^ to −1.5840 kJ·mol^−1^ and bond lengths ranging from 3.192 Å to 3.742 Å. The studies for several phospholipids with short hydrocarbon chains show that changes in chain length have almost no effect on interaction energy, bond length, and partial atomic charge.

## 1. Introduction

Liposomes, spherical vesicles, consist of a phospholipid bilayer that surrounds an aqueous medium and forms an aqueous compartment within [1]. Phospholipids are amphiphilic lipids with a hydrophilic (polar) headgroup and two hydrophobic (nonpolar) hydrocarbon tails [2]. Phospholipid headgroup can be zwitterionic, positively or negatively charged, while the hydrocarbon tail can vary in length and degree of unsaturation [3, 4]. Due to its amphiphilicity, a liposome made of phospholipid can encapsulate polar drugs such as vitamin C in their aqueous compartment and nonpolar drugs such as beta-carotene in the lipid bilayers of the liposome membrane [5–8]. The liposome-based drug delivery system works by delivering drugs directly to the active site (targeted delivery); thus, it is crucial to maintain the stability of the liposome membrane and the drug contained within it so that the drug may reach the target cell [9, 10]. Adding cholesterol to the liposome assembly process makes it possible to maintain and even increase the membrane's stability. An adequate amount of cholesterol in liposomes can decrease membrane permeability and increase membrane stiffness, enhancing the membrane's stability [11–15].

The liposomes used in drug delivery systems have been studied experimentally and computationally for polar and nonpolar drugs [12–21]. Computational studies on issues related to experimental studies can confirm or complement relevant data or information acquired experimentally or even simulate trials to conduct future experiments more efficiently. Computer simulations can provide a better understanding of chemical phenomena, such as the formation of intermolecular interactions, that are difficult to prove by experimentation only [22]. Intermolecular interactions play an essential role in chemical processes [23]. Intermolecular interactions, particularly noncovalent interactions, such as hydrogen bonds and Van der Waals interactions, play an essential role in the design of drug delivery system models. An ideal design will improve the efficacy of the drug delivery process from the moment it enters the body until it reaches the targeted cell. Drugs not covalently bound to their carrier are known to be less harmful to body cells than those covalently bound to their carrier. Therefore, to design an optimum drug delivery system, it is necessary to understand these interactions, which may be analyzed more efficiently using computational chemistry [24, 25].

Drug encapsulation in phospholipid-based liposomes represents one of the most promising future drug delivery system technologies. In the context of computational studies of liposomes, there are still limited studies examining the interactions between every component in the liposomal system. This study aims to better understand the interactions between drug molecules and liposome components by employing an *in silico* approach. We also study the effect of phosphatidylcholine tail length on several interaction parameters, such as interaction energy, bond length, type of interaction, and partial atomic charge. The findings confirm that interaction amongst molecules in the drug-liposomal structures is indeed noncovalent, which in the long run, will facilitate the delivery process of the drug itself.

## 2. Methods

This *in silico* study used phosphatidylcholine (PC), cholesterol, and two active compounds with opposite polarities: polar vitamin C and nonpolar beta-carotene. We investigated the parameters such as optimization energy, interaction energy, bond length, type of interaction, and partial atomic charge. The phosphatidylcholine with a choline head group and hydrocarbon tails of various lengths were: 4 carbon atoms [PC(C4)], six carbon atoms [PC(C6)], eight carbon atoms [PC(C8)], and ten carbon atoms [(PC(C10)]. The software includes NWChem 6.3 [26] for geometry optimization and interaction energy estimation, Notepad^++^ for coordinate structure arrangement, and Chemcraft [27] for molecular visualization.

The research began with the optimization of molecular geometry to obtain each molecule's most stable conformational structure indicated by its lowest energy (*E*_min_). After that, the initial interaction was carried out on two desired molecules to get the position and distance between both molecules, which gave a relatively low interaction energy. Based on these data, further interaction was carried out for each pair of molecules as follows: (i) phospholipid-cholesterol interactions by interacting the phospholipid's [PC(C4)] phosphate group (H40 atom) with the cholesterol's hydroxyl group (O60 atom) at their optimum initial interaction distance, 1.75 Å; (ii) phospholipid-beta-carotene interactions by interacting the phospholipid's [PC(C6)] methyl group (H68 atom) with the beta-carotene's methyl group (C110 atom) at their optimum initial interaction distance 2.00 Å; (iii) beta-carotene-vitamin C interactions by interacting beta-carotene's methyl group (C9 atom) with the vitamin C's hydroxyl group (H97 atom) at 2.00 Å; (iv) cholesterol-vitamin C interactions by interacting the cholesterol's hydroxyl group (O1 atom) with the vitamin C's hydroxyl group (H88 atom) at 1.75 Å; (v) phospholipid-vitamin C interactions by interacting the PC(C4)'s phosphate group with the vitamin C's hydroxyl group at a distance of 1.75 Å, between the O9 atom of phospholipid with the H73 atom of vitamin C and the H40 atom of phospholipid with the O60 atom of vitamin C.

Besides PC(C4), we also used PC(C6), PC(C8), and PC(C10) to investigate the influence of different tail lengths on the interaction properties. The point of interaction was as follows: (i) PC(C6), the O9 atom of phospholipid with the H85 atom of vitamin C and the H40 atom of phospholipid with the O72 atom of vitamin C; (ii) PC(C8), the O9 atom of phospholipid with the H97 atom of vitamin C and the H40 atom of phospholipid with the O84 atom of vitamin C; (iii) PC(C10), the O9 atom of phospholipid with the H109 atom of vitamin C and the H40 atom of phospholipid with the O96 atom of vitamin C.

The interaction energy (*E*_*i*_) between molecules *A* and *B* is determined from the molecular association energy (*E*_*A,B*_) and the sum of the energies of *A* and *B* (*E*_*A*_ + *E*_*B*_) [17, 28, 29], as in the following equation:

(1) $$\begin{array}{c} E_{i}=E_{A,B}-\left(E_{A}+E_{B}\right)\text{.} \end{array}$$

For example, in the calculation of the interaction energy of PC(C4) with vitamin C, *E*_*A*_ represents the molecular energy of PC(C4), *E*_B_ represents the molecular energy of vitamin C and *E*_*A,B*_ represents the molecular association energy between PC(C4) and vitamin C (PC(C4)⋯VitC).

## 3. Results and Discussion

### 3.1. Geometry Optimization

**Figure 1:**
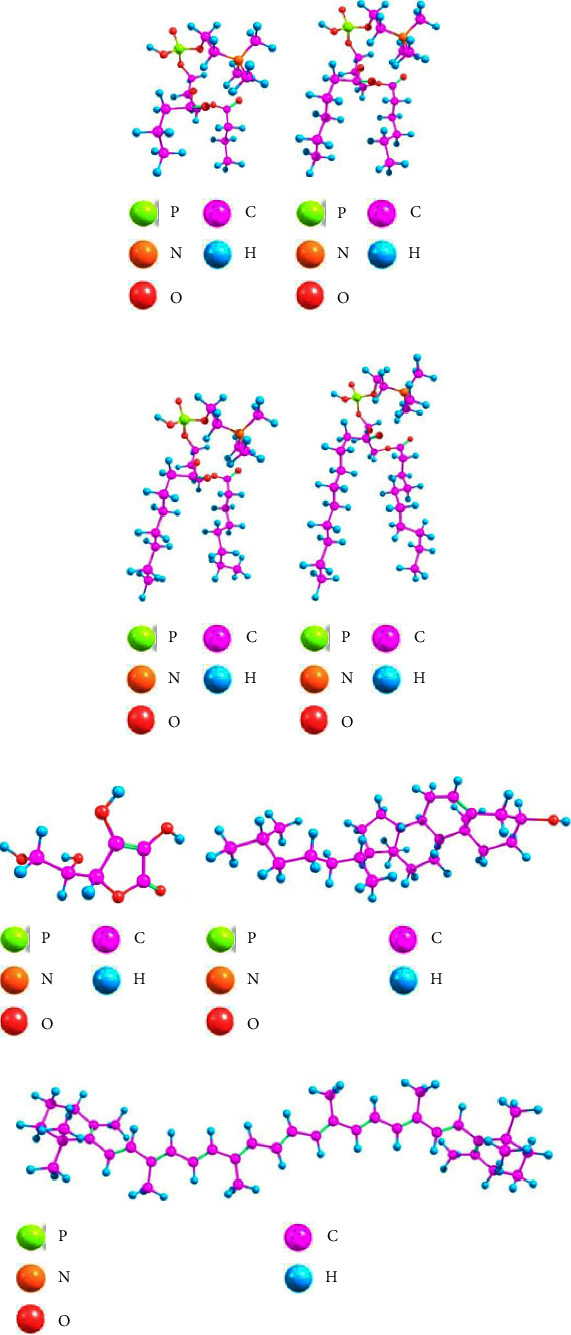
Optimized geometric structure of molecules: (a) PC(C4), (b) PC(C6), (c) PC(C8), (d) PC(C10), (e) vitamin C, (f) cholesterol, and (g) beta-carotene.

Geometry optimization is a method of predicting the three-dimensional arrangement of atoms within a molecule's space. By computing the bond length and angle with the lowest steric resistance, this procedure will determine the conformation of a molecule with the lowest energy. Bond length is the distance between the nuclei of two bound atoms, whereas bond angle is the angle between two neighbouring atoms in a molecule. Both are essential parameters for determining a molecule's geometry. Before searching for a new conformation with lower energy, we determined the initial geometry's energy. Therefore, this method will require rotating the atomic positions and doing energy calculations for each position until the minimum total energy is achieved. The minimum energy of a molecule concerning atomic coordinates shows that geometry optimization leads to the most stable conformation of the related molecule [30–32].  Figure 1 shows the best geometric arrangement or structure of each molecule used in this study, while Table 1 shows the minimum optimization energy.

Table 1 indicates that all molecules' minimum optimization energy (*E*_min_) is negative, indicating that the attractive force is stronger than the repulsive force. When the constituent atoms of a molecule interact with one another to build a stable structure, intramolecular interactions (also called intramolecular forces) occur. Essentially, the two forces work together continuously to shift the atoms in a molecule closer and further apart. However, the most stable bonds can form spontaneously if the attractive forces are strong enough to balance the repulsive forces (the net force is zero; hence the system's potential energy is minimum) [23, 33]. Compared to the data in Figure 1 and Table 1, the required minimum optimization energy decreases as the molecular structure's size or bulkiness increases. The large molecule with several constituent atoms and lengthy chains has weak intramolecular interactions, requiring less energy to break and establish bonds during the optimization process.

After the geometry optimization, we conducted interaction modelling. In this study, interaction modelling was conducted by interacting with the atoms of two different molecules at a certain distance to determine how these molecules would interact. Each interaction model will have a distinct initial distance, as this is the optimum distance for two interacting molecules to have a stable interaction with low initial interaction energy. The lower the interaction's energy, the more stable and favourable it is [23].

### 3.2. Phospholipid Interaction with Cholesterol

**Figure 2:**
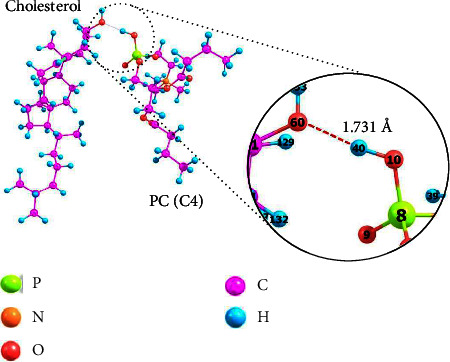
The interaction of phospholipid and cholesterol molecules using an optimized structure.

Adding a certain amount of cholesterol with a rigid structure can increase the stability and decrease the liposome membrane permeability (leakage rate) used in a drug delivery system.  Figure 2 shows a model of how phospholipids and cholesterol interact.

In this model, [PC(C4)], the phospholipid with the shortest hydrocarbon chain length, was chosen to speed the formation of liposome membranes. Besides, liposomes made of phospholipids with shorter chains will have a less rigid membrane and more excellent permeability compared to those with longer chains, making it easier to study the interaction and effect of cholesterol on liposomes. According to Figure 2, the interaction is formed through the active site of the phospholipid phosphate group and the cholesterol hydroxyl group with interaction energy (PC(C4)⋯Cholesterol) of −65.6775 kJ·mol^−1^ and a bond length (H_40_⋯O_60_) of 1.731 Å. The interaction is a hydrogen bond of moderate strength based on the interaction's energy and the bond's length. The moderate strength is because noncovalent interactions, especially hydrogen bonds of moderate strength, have interaction energy of less than 20 kJ·mol^−1^ and bond lengths ranging from 1.5 to 3.0 Å [17, 34, 35]. A hydrogen bond is formed when dipole-dipole interaction occurs between a hydrogen atom polarly bonded to an electronegative atom such as N, O, or F. As an intermolecular force, hydrogen bonds are often stronger than ordinary dipole-dipole, dispersion, and Van der Waals forces but weaker than covalent and ionic bonds. The conformation and 3D structure of biomolecules are attributed to noncovalent hydrogen bonds due to their ability to create dependable and directed bindings [24, 36, 37].

The total charge of a molecule is equal to the sum of each atom's partial charge [38]. The partial atomic charge reflects charge density distribution within molecules [39] or chemical bonds.  Table 2 shows the changes in the partial atomic charges of the phospholipid and cholesterol atoms before and after the interaction. Since the O_60_ atom of the cholesterol hydroxyl group is more electronegative than the H_40_ atom of the phospholipid phosphate group, the O_60_ atom has a negative partial charge. In contrast, the H_40_ atom has a positive partial charge. In its interaction with phospholipids, the electronegative property of the O_60_ atom of cholesterol causes the partial atomic charge to become more negative because it has a more significant potential to attract electrons. In contrast, the H_40_ atom of phospholipids becomes more positively charged. There was a change in the partial atomic charge, but as the change was small, the conformational structure of each molecule remained stable [40]. Encapsulation does not result in a chemical change when phospholipids and cholesterol interact by forming hydrogen bonds, which is physical interaction. This evidence confirms that adding cholesterol to increase the rigidity of liposomes while keeping their fluidity and decreasing their permeability to improve encapsulation efficiency is acceptable, as cholesterol does not cause chemical changes to the liposomes themselves [13–16, 18, 21, 41].

### 3.3. Phospholipid Interaction with Beta-Carotene

**Figure 3:**
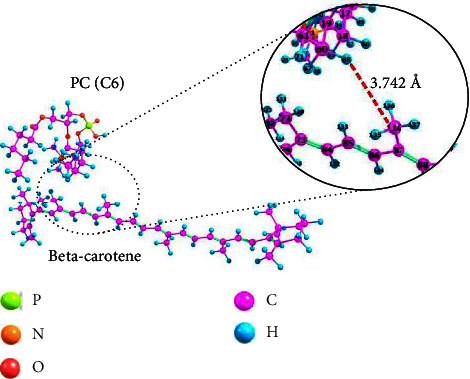
The interaction of phospholipid and beta-carotene molecules using an optimized structure.

Nonpolar beta-carotene is one of the drugs encapsulated by liposomes in this study. We used [PC(C6)] to model the interaction between phospholipids and beta-carotene.  Figure 3 shows how phospholipids interact with beta-carotene.

Interactions occurred between the nonpolar part of phospholipids, the hydrocarbon chain, and beta-carotene itself. This result is consistent with previous studies [13, 14, 41], which showed that liposomes could encapsulate nonpolar drugs in their membranes, specifically between the phospholipid bilayers dominated by hydrophobic hydrocarbon chains. This interaction is a Van der Waals interaction created by the London dispersion force between two nonpolar molecules (induced dipole-induced dipole). The London dispersion force is the attractive force between neighbouring nonpolar molecules [42]. The random movement of negatively charged electrons surrounding a positively charged nucleus induced a short-range interaction [43, 44]. If electrons tend to gather at one end of the molecule, the charge distribution at that end will temporarily shift [45]. This shift gives the molecule a short negative dipole, allowing it to induce the opposite dipole on neighbouring nonpolar molecules, resulting in an attraction interaction by forming an induced dipole-induced dipole [43]. The interaction energy (PC(C6)⋯beta-carotene) is −4.4735 kJ·mol^−1^, and the bond length (H_68_⋯C_110_) is 3.742 Å. Van der Waals interactions between these two molecules, a weak noncovalent interaction [43], allow beta-carotene to be quickly released from liposomes when it reaches the target cell.

### 3.4. Beta-Carotene Interaction with Vitamin C

**Figure 4:**
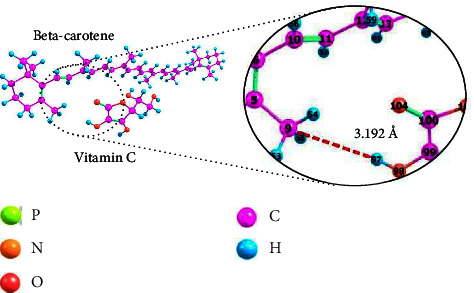
The interaction of beta-carotene and vitamin C molecules using an optimized structure.

In studying the interactions between liposome components as a drug delivery system, the interactions between encapsulated drugs should not be neglected, especially if the drugs encapsulated in the same liposome have different properties. Therefore, this study also looked into the interaction between beta-carotene, a nonpolar drug, and vitamin C, a polar drug, encapsulate.  Figure 4 illustrates the interaction between the two previously stated drugs.

The presence of an induced dipole-dipole reveals that the interaction between these two molecules is a Van der Waals interaction. The induced dipole-dipole interaction occurs when vitamin C, which is polar and has a permanent dipole, induces beta-carotene, which is initially nonpolar and without a dipole, to have a momentary or an induced dipole [46, 47]. Since only Van der Waals interactions exist between beta-carotene and vitamin C, it can be said that there will be no chemical changes between these two drugs if they are simultaneously encapsulated within one liposome. Beta-carotene interacts with the phospholipid tail (see Figure 4), while vitamin C interacts with the phospholipid head group (see Figure 6), allowing simultaneous encapsulation without interfering. Van der Waals interaction, on the other hand, as weak noncovalent interaction, allows beta-carotene and vitamin C to be released from liposomes once they reach their target cells in the body [43]. According to computational calculations, the interaction energy (beta-carotene⋯VitC) is −1.5840 kJ·mol^−1^, and the bond length (C9⋯H97) is 3.192 Å.

### 3.5. Cholesterol Interaction with Vitamin C

Nonpolar cholesterol will be encapsulated between the liposome bilayer, which is dominated by phospholipid hydrocarbon chains, whereas polar vitamin C will be encapsulated in the aqueous compartment of the liposome. Therefore, it can be assumed that these two molecules occupy distinct parts within a liposome.

**Figure 5:**
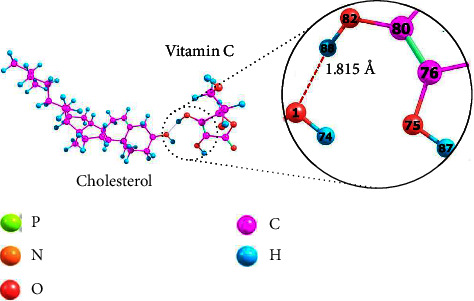
The interaction of cholesterol and vitamin C molecules using an optimized structure.

Figure 5 depicts the modelling of the interaction between these two molecules to determine whether it is true that cholesterol and vitamin C in the same liposome does not affect each other. The interaction occurs through the active site of the cholesterol hydroxyl group and the vitamin C hydroxyl group, with interaction energy (cholesterol⋯VitC) of −45.6734 kJ·mol^−1^ and a bond length (O_1_⋯H_88_) of 1.815 Å. These two values indicate that the interaction is a hydrogen bond of moderate strength [34].

The interaction between cholesterol and vitamin C causes changes in the partial atomic charge of the two molecules. However, the difference is relatively small, indicating that the conformational structure of the molecule remains stable [40].  Table 3 displays the difference in the partial atomic charges of cholesterol and vitamin C atoms before and after the interaction. Before the interaction, the O_1_ atom of cholesterol was partially negatively charged, whereas the H_88_ atom of vitamin C was partially positively charged, as the O_1_ atom was more electronegative than the H_88_ atom. Due to its tendency to attract electrons when interacting, the O_1_ atom becomes more negatively charged after the interaction has occurred. Because the interaction between the two is physical, there will be no chemical interaction. This result supports the fact that adding the appropriate amount of cholesterol to liposomes can improve the encapsulation efficiency of vitamin C as an encapsulated polar drug [13–16, 18].

### 3.6. Phospholipid Interaction with Vitamin C

**Figure 6:**
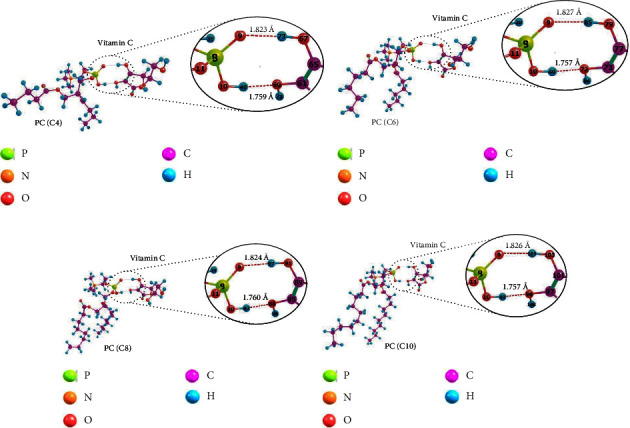
The interaction of phospholipid and vitamin C molecules using an optimized structure, as follows: (a) PC(C4)⋯VitC, (b) PC(C6)⋯VitC, (c) PC(C8)⋯VitC, and (d) PC(C10)⋯VitC.

Similar studies on the interaction between phospholipids and vitamin C using the same type of phospholipid have been conducted on the hydrocarbon chain lengths (C4) and (C20) [17]. This study also utilized a wider variety of hydrocarbon chain lengths, such as (C4), (C6), (C8), and (C10), with vitamin C as a polar drug encapsulated in liposomes. Figure 6 shows the modelling of the interaction between each variation of phospholipid and vitamin C. The two interact through the active site of the phospholipid phosphate group and the hydroxyl group of vitamin C to form two hydrogen bonds between the O and H atoms.

All data relevant to Figure 6 are in Table 4. Similar to earlier research [17], it was proven that the interaction between phospholipids and vitamin C is a moderately strong hydrogen bond. This interaction also allows the release of vitamin C from liposomes when it reaches the target cells in the body. The hydrogen bonds formed here are also cooperative because the interaction energy (*E*_*i*_) obtained is the sum of the energies of all hydrogen bonds formed [48, 49]. Cooperative hydrogen bonds are known to decrease bond strength [49]. As evidenced by the data in Table 4, phospholipids with shorter hydrocarbon chains have lower interaction energy, indicating a weaker bond than phospholipids with longer hydrocarbon chains, although the difference is insignificant. Overall, the length variation of the phospholipid hydrocarbon chain used in this study had no significant impact on bond length or interaction energy of the phospholipids⋯vitamin C, as evidenced by the slight difference in value.

The partial charges of the phospholipid and vitamin C atoms that interact are given in Table 5. The findings indicate that the length variation of the phospholipid hydrocarbon chain does not significantly affect the partial charges of the interacting atoms. The findings thus far indicate that there is no chemical change in any component of the liposome or the encapsulated drug; however, the physical interactions that are formed facilitate the release of drugs from liposomes.

## 4. Conclusion

*In silico* studies of the interactions in a liposome-based drug delivery system confirmed that the interactions between phospholipids, cholesterol, beta-carotene, and vitamin C were noncovalent, moderate-strength hydrogen bonds and Van der Waals interactions. The moderate-strength hydrogen bonds have interaction energies ranging from −73.6434 kJ·mol^−1^ to −45.6734 kJ·mol^−1^ and bond lengths ranging from 1.731 Å to 1.827 Å. In contrast, Van der Waals interactions have interaction energies ranging from −4.4735 kJ·mol^−1^ to −1.5840 kJ·mol^−1^ and bond lengths ranging from 3.192 Å to 3.742 Å. Only physical bonds in every noncovalent interaction are formed, with no chemical interaction. The results imply that using phospholipids as the raw material for liposomes will not cause chemical changes in other molecules or drugs encapsulated within them. Weak noncovalent interactions also imply that drug release from liposomes will be straightforward once the drug reaches its target cells in the body. This study also reveals that differences in hydrocarbon chain length have negligible impact on interaction energy, bond length, and partial atomic charge.

## Figures and Tables

**Table 1:** The minimum optimization energy (*E*_min_) of the optimized molecule.

Molecule	*E*_min_ (kJ·mol^−1^)
PC(C4)	−6774.7550
PC(C6)	−7428.0841
PC(C8)	−8081.3927
PC(C10)	−8734.7209
Vitamin C	−2849.4701
Cholesterol	−4703.3064
Beta-carotene	−6473.9095

**Table 2:** The phospholipid^*∗*^ and cholesterol^*∗∗∗*^ atoms' partial atomic charges before and after the interaction.

Atoms		Partial atomic charges
Before interaction	H_40_^_*∗*_^	0.38
	O_60_^_*∗∗∗*_^	−0.66

After interaction	H_40_^_*∗*_^	0.45
	O_60_^_*∗∗∗*_^	−0.72

**Table 3:** The cholesterol^*∗∗∗*^ and vitamin C^*∗∗*^ atoms' partial atomic charges before and after the interaction.

Atoms		Partial atomic charges
Before interaction	O_1_^*∗∗∗*^	−0.66
	H_88_^*∗∗*^	0.37

After interaction	O_1_^*∗∗∗*^	−0.73
	H_88_^*∗∗*^	0.42

**Table 4:** Phospholipid and vitamin C interaction parameters.

Interacted molecules	Interacted atoms	*E*_*i*_ (kJ·mol^−1^)	Bond length (Å)	Type of interaction
PC(C4)⋯VitC	O_9_⋯H_73_	−73.6434	1.823	Hydrogen bond of moderate strength
	H_40_⋯O_60_		1.759	

PC(C6)⋯VitC	O_9_⋯H_85_	−73.6259	1.827	Hydrogen bond of moderate strength
	H_40_⋯O_72_		1.757	

PC(C8)⋯VitC	O_9_⋯H_97_	−73.0003	1.824	Hydrogen bond of moderate strength
	H_40_⋯O_84_		1.760	

PC(C10)⋯VitC	O_9_⋯H_109_	−72.5849	1.826	Hydrogen bond of moderate strength
	H_40_⋯O_96_		1.757	

**Table 5:** Partial atomic charges in the region of interaction between phospholipid^_*∗*_^ and vitamin C^_*∗∗*_^.

Interacted molecules	Atoms	Partial atomic charges
PC(C4)⋯VitC	O_9_^_*∗*_^	−0.75
	H_73_^_*∗∗*_^	0.43
	H_40_^_*∗*_^	0.45
	O_60_^_*∗∗*_^	−0.74

PC(C6)⋯VitC	O_9_^_*∗*_^	−0.76
	H_85_^_*∗∗*_^	0.43
	H_40_^_*∗*_^	0.45
	O_72_^_*∗∗*_^	−0.74

PC(C8)⋯VitC	O_9_^_*∗*_^	−0.76
	H_97_^_*∗∗*_^	0.43
	H_40_^_*∗*_^	0.45
	O_84_^_*∗∗*_^	−0.74

PC(C10)⋯VitC	O_9_^_*∗*_^	−0.76
	H_109_^_*∗∗*_^	0.43
	H_40_^_*∗*_^	0.45
	O_96_^_*∗∗*_^	−0.74

## Data Availability

The data used to support the findings of this study are included within the article. Samples of the compounds are available from the authors.
